# Embelin Protects Against Acute Pentylenetetrazole-Induced Seizures and Positively Modulates Cognitive Function in Adult Zebrafish

**DOI:** 10.3389/fphar.2019.01249

**Published:** 2019-10-25

**Authors:** Uday Praful Kundap, Brandon Kar Meng Choo, Yatinesh Kumari, Nafees Ahmed, Iekhsan Bin Othman, Mohd Farooq Shaikh

**Affiliations:** ^1^Department of Neurosciences, University of Montreal Hospital Centre (CRCHUM), Montreal, Canada; ^2^Neuropharmacology Research Laboratory, Jeffrey Cheah School of Medicine and Health Sciences, Monash University, Kuala Lumpur, Malaysia; ^3^School of Pharmacy, Monash University Malaysia, Kuala Lumpur, Malaysia

**Keywords:** embelin, epilepsy, cognitive disorder, T-maze behavior, zebrafish

## Abstract

**Purpose of the research:** Epilepsy is a continuous process of neurodegeneration categorized by an enduring tendency to generate uncontrolled electrical firing known as seizures causing involuntary movement all over the body. Cognitive impairment and behavioral disturbances are among the more alarming co-morbidities of epilepsy. Anti-epileptic drugs (AEDs) were found to be successful in controlling epilepsy but are reported to worsen cognitive status in patients. Embelin (EMB) is a benzoquinone derived from the plant *Embelia ribes* and is reported to have central nervous system (CNS) activity. This study aims to evaluate the effectiveness of EMB against pentylenetetrazole (PTZ) induced acute seizures and its associated cognitive dysfunction. This was done *via* docking studies as well as evaluating neurotransmitter and gene expression in the zebrafish brain.

**The principal results:** Behavioral observations showed that EMB reduced epileptic seizures and the T-maze study revealed that EMB improved the cognitive function of the fish. The docking study of EMB showed a higher affinity toward gamma-aminobutyric acid (GABA_A_) receptor as compared to the standard diazepam, raising the possibility of EMB working *via* the alpha subunit of the GABA receptor. EMB was found to modulate several genes, neurotransmitters, and also neuronal growth, all of which play an important role in improving cognitive status after epileptic seizures. Healthy zebrafish treated with EMB alone were found to have no behavioral and biochemical interference or side effects. The immunohistochemistry data suggested that EMB also promotes neuronal protection and neuronal migration in zebrafish brains.

**Major Conclusions:** It was perceived that EMB suppresses seizure-like behavior *via* GABA_A_ receptor pathway and has a positive impact on cognitive functions. The observed effect was supported by docking study, T-maze behavior, neurotransmitter and gene expression levels, and immunohistology study. The apparatus such as the T-maze and seizure scoring behavior tank were found to be a straightforward technique to score seizure and test learning ability after acute epileptic seizures. These research findings suggest that EMB could be a promising molecule for epilepsy induced learning and memory dysfunction.

**Graphical Abstract:**
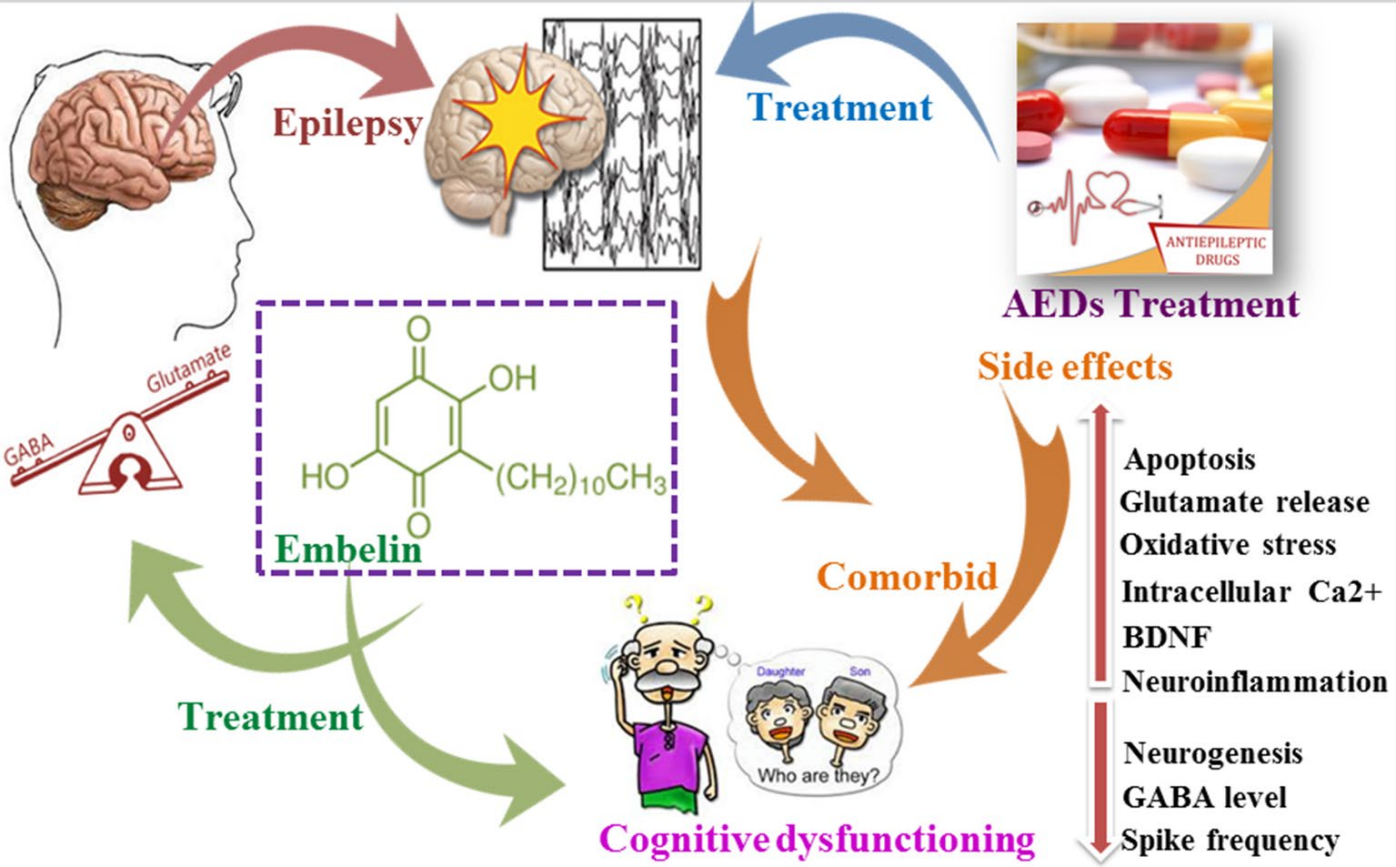
Impact of epilepsy and antiepileptic drugs on cognitive dysfunction.

## Bullet Point Summary:

1. What is already known:
  - Epilepsy related comorbidities such as cognitive dysfunction are common in many people with epilepsy.
  - Most of the anti-epileptic drugs help in preventing seizures but have a negative impact on cognitive functions.
  - A crude extract of the plant *Embelia ribes* is used to treat epilepsy in alternative medicine.
2. What this study adds:
  - Embelin isolated from *Embelia ribes* was found to be effective against seizures and prevented memory decline in zebrafish.
  - Embelin modulates neurotransmitters and gene expression as well as exhibits a neuroprotective effect.
  - A docking study of EMB shows that it has a high affinity toward the gamma-aminobutyric acid (GABA_A_) receptor and possibly works *via* the alpha subunit of the GABA receptor.

## Introduction

Epilepsy is a neurological condition with complications associated with diverse neurobiological and behavioral alterations characterized by recurrent, spontaneous epileptic seizures (Galanopoulou et al., 2012). It is the fourth most common neurological disorder (Newton and Garcia, 2012), affecting over 70 million people of all ages around the world (Copmans et al., 2017). The significant feature of epilepsy are the seizures, but it also affects cognition, leading to a poor quality of life (Van Rijckevorsel, 2006; Kim and Ko, 2016). The prevalence of memory problems in patients with epilepsy is 40–45%, and they experience difficulties in problem-solving and learning as well as have psychomotor retardation (Mula, 2015). A notion exists that the rate/effects of seizures and the dose of AEDs play a key role in the cognitive decline of epileptic patients (Lagae, 2006; Park and Kwon, 2008). Adverse effects due to cognitive impairment are a major problem associated with AEDs as they alter the role of different genes that are associated with epileptogenesis and memory function (Gupta et al., 2017) (Gayatri and Livingston, 2006).

Few studies have investigated the role of genes such as neuropeptide Y (NPY) gene, CREB1, and brain-derived neurotrophic factor (BDNF) that interact with each other to control epilepsy and enhance long term memory (Gøtzsche and Woldbye, 2016; Luo et al., 2017). BDNF is a small protein secreted in the brain that binds to its p75 and TrKB receptors and has a remarkable role in memory, survival, and differentiation of neurons in the brain (Roopra et al., 2012). It has been known that BDNF is linked with the inception of epileptogenesis, and the epileptic condition is avoided by the upregulation of BDNF indicator in the brain (Binder, 2004). The cAMP response element (CRE) is associated with several genes responsible for epileptogenesis in the form of a promoter, and it has been identified to phosphorylate cAMP response to generate epilepsy (Zhu et al., 2012). Theories about the cAMP-response element binding protein (CREB) and memory are still evolving. Study in mice shows that decreased CREB levels have 50% decrease in seizures episode and require more electrical kindle stimulations (Zhu et al., 2012). Earlier study has reported that chronic spontaneous seizures are reduced by NPY in a temporal lobe epilepsy model of rats (Noe et al., 2008). The anticonvulsant effect of NPY gene overexpression in rat brains shows the important role in controlling epilepsy of NPY (Noe et al., 2010). NPY has been recently proposed for gene therapy, depending upon patient data, to control epilepsy using various viral vector carriers. These trials were successful in various transgenic rodent models (Sørensen and Kokaia, 2013).

In recent years, the primary focus has turned toward non-mammalian epilepsy models for behavior testing, due to numerous factors. These factors including greater cost-effectiveness, high genetic correlation with humans, and rapid breeding (Arief et al., 2018). The cost and time required to carry out gene expression and brain cell genesis studies in zebrafish are less as compared to rodents (Mussulini et al., 2013). The main limitation in executing research in epilepsy and cognitive abnormalities is the lack of precise and reproducible animal models for the testing of new drug molecules (Yam Nath Paudel et al., 2018). To overcome this limitation, our laboratory has developed a zebrafish model of epilepsy induced cognitive dysfunction and confirmed the hypothesis that both epilepsy and AEDs affect cognitive functions in zebrafish (Kundap et al., 2017b). Although a study is conducted using zebrafish, the findings can still be translated to mammals, particularly humans, as over 70% of zebrafish genes are substantially similar to their mammalian orthologues (Macrae and Peterson, 2015). Another reason is that 70% of zebrafish genes also have one or more human orthologues, and 47% of human genes have a one to one relationship with a corresponding zebrafish gene (Howe et al., 2013). There are also studies that have established that zebrafish brain regions show homologous functions to their mammalian counterparts, despite the substantial neuroanatomical differences (Fontana et al., 2018). Recent studies have shown that many different genetically modified zebrafish models (Samarut et al., 2018) are used as initial *in vivo* screening tools for compounds derived from natural sources (Samarut et al., 2019).

Numerous natural products, including derivatives of quinone which are thought to have better efficacy and safety profiles, are known for their central nervous system (CNS) related activity (Durg et al., 2017). Embelin (EMB) exhibits favorable chemical and physical properties, and its capacity for penetrating the blood–brain barrier (BBB) (Bhuvanendran et al., 2018) makes it a suitable candidate for the treatment of CNS related complications (Pathan et al., 2009). EMB is a water-insoluble compound with a LogP value of 4.71 (octanol–water) (Xu et al., 2005), meaning that the compound is highly lipophilic and can reach the brain to exert its effect. Phytochemical and pharmacological investigations discovered that the presence of EMB is a vital element in treating CNS disorders (Kundap et al., 2017a).

Therefore, the primary goal of this study was to evaluate the effectiveness of EMB against pentylenetetrazole (PTZ)-induced seizures and associated cognitive dysfunction using adult zebrafish as an animal model. In this study, we tried to use a simple T-maze apparatus to measure the memory function of the fish after a single administration of PTZ at an acute dose. We hypothesized that EMB may work by modulating several genes that cause epilepsy, the levels of GABA, and also possibly *via* the GABA_A_ receptor as it has a higher affinity for the receptor as compared to the standard drug diazepam. In spite of the proven role of EMB in epilepsy related genes such as NPY, BDNF, and cAMP-responsive element-binding protein 1 (CREB_1) genes (Kundap et al., 2017a), we attempted to elucidate the overall activity of EMB to support our hypothesis by using molecular docking, immunohistochemistry, and pharmacological, biochemical, and behavioral experimentations. Exploring the cellular and molecular mechanism of compounds from natural sources against epilepsy induced cognitive dysfunction will pave the way for further research and can be a potential alternative to mainstream AEDs.

## Techniques and Materials

### Experimental Equipment and Chemical List

All reagents used were analytical grade unless specified otherwise. Water was purified and filtered with a specific LC-MS filter using a Milli-Q system from Millipore (Bedford, MA, USA). Glutamate (Glu), gamma-amino butyric acid (GABA), acetylcholine (Ach), diazepam (DZP), PTZ, paraformaldehyde (PFA), phosphate buffered saline (PBS), benzocaine (BZ), and bromodeoxyuridine (BrdU) were purchased from Sigma–Aldrich (USA). Ethanol 95% (EtOH) was purchased from KOLIN Chemicals Co. Ltd., Korea, methanol (MeOH), chloroform (CHCl3), isopropanol (IPA), and formic acid (FA) were purchased from Friedemann Schmidt Chemicals, Parkwood 6147, Western Australia. The pure form of plant extract of EMB was purchased from YUCCA Enterprises, Mumbai, India (purity—97.90%, moisture—1.90%). Fish tanks (10 L capacity) were purchased from Petco Pet Keeper, Malaysia. The Sony Handycam (AVCHD 5x) recorder, Sony Camcorder stand, Smart 3.0.05 Tracking Software (Panlab, Harvard Apparatus), Hamilton Syringe 700–702 series 25 µl, BD Disposable Needle (30G), FSC 22 Frozen Section Media (Leica Biosystems, Nussloch, Germany), Leica CM1860 Cryostat (Leica Biosystems, Nussloch, Germany), and Agilent Infinity 1290 UHPLC, coupled with Agilent 6410 Triple Quad LC/MS and the Applied Biosystems StepOnePlus™ Real-Time PCR Systems, were also used in this work.

### Zebrafish (*Danio rerio*) Care and Maintenance

Adult zebrafish (*Danio rerio*) of heterozygous wild-type-AB stock (standard short-fin phenotype) were obtained from the Institute of Molecular and Cell Biology (IMCB), 61 Bioplis Drive Proteos, Singapore 138673. All fish were kept in the Monash University Malaysia fish facility at 28°C, with a 10/14 h dark/light cycle (white incident light off at 10 pm, white incident light on at 8 am) under aquarium conditions. Care was taken to maintain the system water pH between pH 6.8 and 7.1 by using an electronic pH pen (Classic PH Pen Tester, Yi Hu Fish Farm Trading, Singapore 698950), and the intensity of light was maintained uniformly all over the housing area at 250 Lux. Fish were nourished with TetraMin^®^ Tropical Flakes twice a day and livestock of Artemia from Bio Marine (Aquafauna, Inc. USA) once a day to ensure a constant source of the food supply with *ad libitum* feeding. Zebrafish tanks with dimensions of 36 cm x 26 cm x 22 cm were used to house the fish, and the tanks were equipped with a water circulation system for constant aeration (Kundap et al., 2017b). Group housing was used, whereby 10–12 fishes/tank, males, and females were housed separately. The Monash Animal Research Platform (MARP), Australia, approved all the zebrafish experimental procedures (MARP-2015-084).

### Drug Treatment and Groups

**Figure 1:**
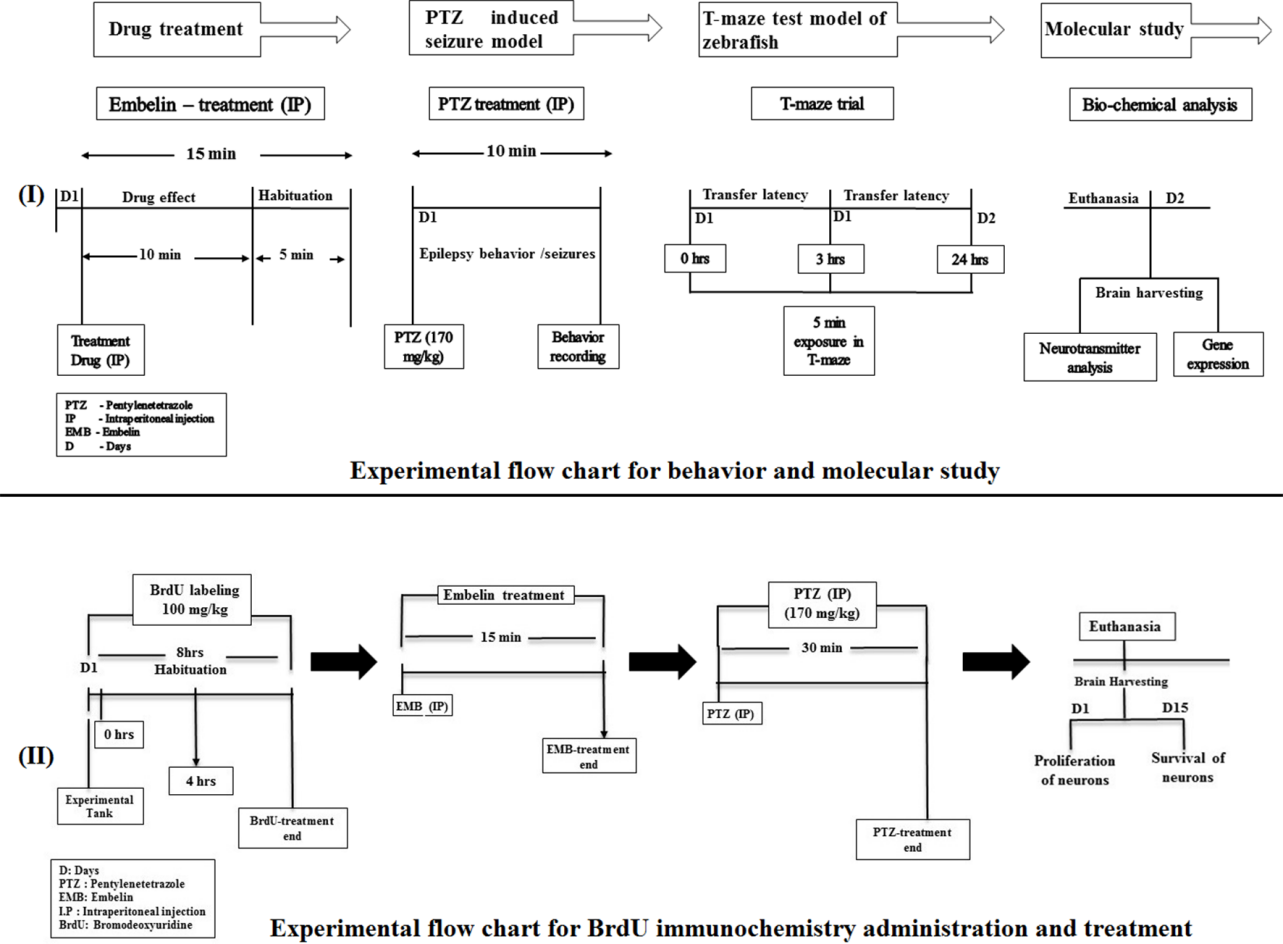
Experimental setup and design procedure. The flowchart represents the scheme for drug treatment, PTZ administration, and behavior recording for epilepsy and T-maze used in the study.

The treatment group of animals were initially injected with the vehicle/test drug and then injected with PTZ to check their seizure behavior. Later, their cognitive status was examined using a T-maze. EMB and standard DZP were dissolved in 10% dimethyl sulfoxide (DMSO). The animals were divided into the following groups, with each group having n = 10 unless mentioned otherwise as shown in **Figure 1**.

#### Set-1 (Only Emb Treatment)

Group I: vehicle control (VHC—10% DMSO) + distilled water; group II: DZP 1.25 mg/kg + distilled water: group III: EMB-0.156 mg/kg + distilled water; group IV: EMB-0.312 mg/kg + distilled water; group V: EMB-0.625 mg/kg + distilled water

#### Set-2 (Epilepsy Behavior Test)

Group I: vehicle control (VHC—10% DMSO) + distilled water; group II: VHC + PTZ 170 mg/kg; group III: DZP 1.25 mg/kg + PTZ (170 mg/kg); group IV: EMB-0.078 mg/kg + PTZ (170 mg/kg); group V: EMB-0.156 mg/kg + PTZ (170 mg/kg); group VI: EMB-0.312 mg/kg + PTZ (170 mg/kg); group VII: EMB-0.625 mg/kg + PTZ (170 mg/kg); group VIII: EMB-1.25 mg/kg) + PTZ (170 mg/kg); group IX: EMB-2.5 mg/kg + PTZ (170 mg/kg); group X: EMB-5 mg/kg + PTZ (170 mg/kg); group XI: EMB-10 mg/kg + PTZ (170 mg/kg)

#### Set-3 (Brdu Immunochemistry)

Group I: vehicle control (10% DMSO); group II: VHC + PTZ (negative control group); group III: EMB 0.156 mg/kg + PTZ (170 mg/kg); group IV: EMB 0.625 mg/kg + PTZ (170 mg/kg)

All the drugs were administered *via* intraperitoneal injection as per the procedure described previously by Kundap et al. (2017b).

#### Procedure for a Zebrafish Anesthesia and Intraperitoneal Injection

PTZ and EMB were intraperitoneally infused into the zebrafish as specified by the protocol given by Kundap et al. (2017b) and is given beneath. When different intraperitoneal infusions were required, the infusions were given at alternating lateral ends, instead of the midline between the pelvic fins.

Each zebrafish was caught individually using a fish holding net and after that moved into an anesthesia preparation (30 mg/L BZ). The fish were kept in the anesthesia water for 30 s until they stop moving. The zebrafish were taken out once anesthetized and weighed afterward to calculate the dose and subsequently the infusion volume. A delicate sponge roughly 20 mm in height was soaked with water and set inside a 60 mm Petri dish. A cut between 10 and 15 mm was made in the sponge to control and hold the fish for the intraperitoneal infusion. The intraperitoneal infusion was given while utilizing a dissection microscope by embedding the needle into the midline between the pelvic fins. An appropriate volume was then injected into the zebrafish, after considering the body weight of the zebrafish.

All intraperitoneal infusions were administered into the stomach pit at an area midline to the pelvic fins, utilizing a 10 μl Hamilton syringe (700 series, Hamilton 80400) (Stewart et al., 2011). The experiment was performed in a separate behavior room, with the room temperature kept between 26 and 30°C and humidity between 50 and 60%. All zebrafish were acclimatized in the said behavior room for 2 h prior to experiment to minimizing any novel tank response. Other precautions taken include using a small injection volume of 10 μl per gram of fish and using a 35-gauge needle. The zebrafish were restrained in a water saturated sponge under BZ anesthesia to reduce the distress inflicted on the zebrafish (Júnior et al., 2012). This intraperitoneal injection technique was found to be effective in zebrafish (Kundap et al., 2017b) and did not cause any mortality throughout the experiment. After the intraperitoneal injection, the zebrafish was immediately transferred to an observation tank.

### Zebrafish Behavior Study for Epilepsy and Cognition

#### Dose Determination Study for Embelin

A wide range of EMB doses were tested, which were from 0.078 to 10 mg/kg. The zebrafish were divided into 11 groups containing 12 fish in each group. DZP was used as a positive control, and the fish received a 1.25 mg/kg dose. The vehicle-treated group received 10% DMSO, followed by a distilled water injection. To induce epileptic seizures, all the animals were first treated individually with the vehicle/DZP/EMB and then injected with 170 mg/kg of PTZ. The resulting seizure behavior was observed for seizure scoring for a period of 10 min post-PTZ injection. The seizure score was used as a parameter to determine the EC_50_ value of the EMB (Vincent, 2010). A graph was plotted as the log of drug concentration against the percentage (%) effect of the drug, giving a familiar sigmoidal shaped graph, to select the effective dose of EMB (Mensor et al., 2001).

#### Epilepsy Behavior and Seizure Score Recording

Adult zebrafish were tested in an observation tank, with the seizure intensity being measured using a special scoring system as per a previously developed protocol (Kundap et al., 2017b). Seizure score, seizure onset time (min), total distance traveled (cm), as well as time spent (s) in upper and the lower halves of the tank were noted. After seizure score analysis, the fish were tested for their cognitive ability using a T-maze test (Banote et al., 2013). Animals administered with a specific PTZ concentration demonstrate different seizure scores, seizure frequencies, seizure profiles, and latencies to reach the seizure scores. Seizures normally last for 10 min after administration of 170 mg/kg of PTZ and progressively decreases with time (Desmond et al., 2012).

#### T-Maze Test

The T-maze is an apparatus which consists of a “T” shaped box containing one straight long arm and two short arms on the left and right-hand side (Wenk, 2001). The right short arm has a bigger opening known as the “deeper chamber,” which acts as a favorable environment for the fish and thus the fish tend to spend a maximal amount of time there. The detailed specifications regarding the maze were as per a previous study. Transfer latencies (TL) were recorded at 0, 3, and 24 h post-PTZ administration. Inflexion ratios (IR3 h) = (L0–L1)/(L1) and (IR24 h) = (L0–L2)/(L2) were calculated, where L0 is the initial latency(s) at 0 h, and L1 and L2 are the latency(s) at the 3 h and 24 h trial, respectively. The behavior recordings during seizure activity and the T-maze test were analyzed to track the locomotor patterns. Tracking of the locomotor pattern was done by using the computer software SMART v3.0—Panlab Harvard Apparatus^®^ (Kundap et al., 2017b).

### Biochemical Estimation

#### Brain Harvesting

The zebrafish brains were harvested at the end of behavior study in order to determine the molecular changes in the brain. The animal groups were divided into two halves, and each brain was then transferred into TRIzol^®^ for gene expression studies and another half into MeOH for LC-MS/MS studies. The whole process of brain harvesting was done under ice-cold conditions, and the brains were immediately transferred into dry ice. All the brains were stored at −80°C until further use.

#### Neurotransmitter Analysis

Glutamate, GABA, and Ach are the significant neurotransmitters in investigating epilepsy and cognition. These neurotransmitters were analyzed using the Liquid Chromatography–Tandem Mass Spectrometry (LC-MS/MS) technique. All the standard neurotransmitters were prepared in MeOH (0.1% FA) as a stock solution of 1 mg/ml and were kept at 4°C until use. Standards for calibration were prepared from the original stock solution. Serial dilutions from 100–2,000 ppb were used for calibration. The brain was homogenized in 200 µl of ice-cold MeOH (0.1% FA). The homogenate was vortex-mixed for 1 min and then centrifuged at 18, 000 × g for 10 min at 4°C. Finally, for LC-MS/MS analysis, the supernatant was pipetted and placed into vials.

LC-MS/MS was run on an Agilent 1290 Infinity UHPLC, coupled with an Agilent 6410 Triple, Quad LC/MS, ZORBAX Eclipse Plus C18 RRHD 2.1 x 150 mm, 1.8-micron (P/N 959759-902) auto-sampler system (Agilent Technologies, Santa Clara, CA, USA). The samples were separated on a SMol-RRHD-Eclipse-C18-8 (15) UHPLC-160129-00011-Pos-DMRM used at 30°C. The mobile phase consisting of 0.1% FA in water (solvent A) and acetonitrile with 0.1% FA (solvent B) was used with a gradient elution: 0–3 min, 50% B; 3–6 min, 95% B; and 06–07 min, 95% B, at a flow rate of 0.1 ml/min. ESI-MS/MS conditions were set as follows: ESI ion source, positive ion polarity, gas temperature 325°C, drying gas flow 9.0 L/min, nebulizer pressure 45 psi, and Vcap 4,000 V. MS acquisition of GABA, Glu, and ACh was performed in electrospray positive ionization multiple reaction monitoring (MRM) mode.

#### Gene Expression

Gene expression studies were carried out for the NPY, BDNF, and cAMP-responsive element-binding protein 1 (CREB_1) genes. All the brain samples were placed in ice-cold 200 µl TRIzol^®^ reagent (Invitrogen, Carlsbad, CA, USA) and immediately stored at −80°C until further usage. The study was divided into three steps, namely, isolation of mRNA, synthesis of cDNA strand, and then real-time PCR to estimate the level of the gene expressed.

#### Isolation of RNA and First Strand cDNA Synthesis

The mRNA was isolated by following the protocol provided by the kit’s manufacturer. In brief, brain tissue was properly homogenized in TRIzol^®^ reagent, mixed with CHCl3, and centrifuged at 13,500 rpm (revolutions per minute) for 15 min at 4°C. The upper aqueous supernatant was transferred into new tubes, and IPA was added, mixed, and incubated for 10 min at room temperature and later centrifuged for 10 min at 13,500 rpm at 4°C. The supernatant was discarded, and the pellets were subjected to rinsing with 75% ethanol. The pellets were air-dried for 5 to 8 min. Finally, nuclease-free water was added to each tube to dissolve the mRNA pellet. The concentration and purity of the isolated mRNA were measured using a NanoDrop Spectrophotometer (Implen NanoPhotometer 190–1,100 nm, Galileo, Madrid, Spain). The mRNA samples were converted into cDNA using the QuantiTect Reverse-Transcription Kit (Qiagen) according to the manufacturer’s protocol.

#### StepOne**^®^** Real-Time PCR

The gene expression of NPY, BDNF, and CREB_1 were measured by real-time quantitative RT-PCR (StepOne Applied Biosystems) using QuantiTect SYBR Green Dye (Qiagen, Valencia, CA). All the primer sets were provided by Qiagen (npy: Dr_npy_1_SG QuantiTect Primer Assay (QT02205763) , bdnf: Dr_bdnf_1_SG QuantiTect Primer Assay (QT02125326), and CREB_1: Dr_CREB_1 bpa_1_SG QuantiTect Primer Assay (QT02197503). The PCR mixture contained 1X SYBR Green PCR Master Mix (Qiagen), 0.7 μM of both forward and reverse primers, and 1 μl of sample cDNA. The samples were incubated at 95°C for 2 min prior to thermal cycling (40 cycles of 95°C for 5 s and 60°C for 15 s). Relative expression values of the above genes were obtained by normalizing the threshold cycle (Ct) values of genes of interest against Ct value of eef1a1b (housekeeping gene) (2 ^ [Ct eef1a1b—Ct gene of interest]).

### Molecular Modeling Studies

All molecular docking studies were performed in BIOVIA Discovery Studio 4.5 (www.accelrys.com). Since the 3D structure of the gamma-aminobutyric acid receptor (GABA_A_ subunit β3) of zebrafish is not available, we performed molecular docking studies of EMB on the human GABA subunit β3 to predict and correlate *in vivo* results. The x-ray crystal structure of the human GABA_A_ subunit β3 complexed with the agonist benzamidine was retrieved from the Protein Databank (PDB code: 4COF) (Miller and Aricescu, 2014). The water molecules were deleted, and hydrogen atoms were added. Finally, the protein was refined with a CHARMM force field at a physiological pH. To validate the docking reliability, a co-crystallized ligand (benzamidine) was first re-docked to the binding site of GABA. Subsequently, DZP and EMB were docked into the same active site, and 30 conformations of each compound were obtained through CDOCKER. The conformation with the lowest energy was selected as the most probable binding conformation for each ligand.

The CDOCKER is CHARMM-based docking algorithm that uses the CHARMM family of force fields and offers all the advantages of full ligand flexibility (including bonds, angles, and dihedrals) and reasonable computation times (Brooks et al., 1983). The CDOCKER algorithm adopts a strategy involving the generation of several initial ligand orientations in the active site of the target protein, followed by molecular dynamics based simulated annealing and final refinement by energy (Mo et al., 2012). The CDOCKER was used for the docking of all compounds. The molecular docking study was carried out to understand the binding mode of EMB within the active site of the GABA_A_ β3 subunit using the Discovery Studio suit 4.5 software.

### Bromodeoxyuridine (BrdU) Immunohistochemistry

#### Mode of Administration

Adult fish were anesthetized in system water tank (1 L) containing 0.016% BZ (pH 7.0; Classic PH Pen Tester, Yi Hu Fish Farm Trading, Singapore 698950). All the fish from each group were individually injected twice with BrdU (100 mg/kg) intraperitoneally (Mao et al., 2009), with a time interval of 4 h. The vehicle/EMB/PTZ was injected intraperitoneally as per the dose described. The animal in the treatment group was injected with the vehicle/EMB doses and habituated for 15 min in an observation tank. To induce epileptic seizures, fish from the EMB and PTZ group were individually exposed to a 170 mg/kg dose of PTZ. The postinjection survival period ranged between 2 h to 15 days, before the brain was extracted for fixation. Later on, after 2 h of PTZ treatment, animals from each group (n = 6) were euthanatized, and brain samples were harvested for immunochemistry studies to check the neuronal loss at day 0. The remaining n = 6 animals were housed separately in the individual tanks for next 15 days to check the survival and the proliferating ability of neurons.

#### Zebrafish Brain Fixation

Immunohistochemistry was performed on the whole brain of adult zebrafish. The animals were deeply anesthetized by immersing them into BZ dissolved in system water. The brain was extracted completely by opening the skull and was fixed overnight at 4°C in 4% PAF in saline phosphate buffer (pH 7.4). The specimen was then soaked into 10% sucrose solution at room temperature until brain sinks to the bottom of the tube (up to 5–6 h). Later, the 10% sucrose solution was replaced with a 20% sucrose solution, and the specimen was soaked overnight at 4°C. Later, the 20% sucrose solution was replaced with a 30% sucrose solution at 4°C and left for up to a week, until used for cryostat sectioning (Ekström et al., 2001).

#### Preparation of Cryostat Brain Section

To prepare cryo-sections, the cryostat machine was set at −20°C (Ekström et al., 2001). Specimens were molded into a cryomold block by using FSC 22 frozen section media inside the cryostat machine at −20°C. The cryostat was set to 20 microns section thickness, and each alternate section was placed on two equally divided glass slides. The sections could adhere to the slide at room temperature for at least 1 h, and later, these slides were stored at −20°C until the immunochemistry procedure.

#### Immunohistochemistry Procedure for the Adult Zebrafish Brain

**Day-1 procedure:** The protocol followed was described earlier by (Malberg et al., 2000). The sections were thoroughly washed three times in PBS with an interval of 5 min. The sections were then immersed in a 50% formamide solution (Vivantis, Inc. USA) for 2 h at 65°C. After 2 h of incubation, the section slides were washed once with 2X-SSC (Sodium Citrate; 0804-4L, Ameresco) for 5 min. Then, sections were incubated in 2N HCl for 30 min at 37°C and washed in a solution of 0.1 M boric acid for 10 min. Blocking solution was added to block the section with 10% normal horse serum (Gibco, Thermo Fisher Scientific, USA) dissolved in PBS containing 0.1% Triton-X-100 (Amresco, Ohio) for 1.5 h. Subsequently, the sections were incubated with mouse anti-BrdU antibody (1:500; Roche Diagnostics, IN, USA) in 10% horse serum with 0.5% bovine serum albumin (BSA) (Sigma Life Science, USA) in PBS containing 0.1% Triton-X-100 for 18 h at 4°C.

**Day-2 procedure:** Sections were washed three times with PBS at the interval of 5 min and incubated for 2 h with the biotinylated horse anti-mouse secondary antibody (1:250; BA-2001, Vector Laboratories, Burlingame, U.S.A). After incubation, the sections were followed by three washes with PBS and incubated with avidin-biotin complex (1:55; VECTASTAIN ABC Kit, Vector Laboratories, CA, U.S.A.) for 2 h at room temperature. After three washes with PBS, the sections were visualized with diaminobenzidine solution (DAB, D4293; Sigma), which was prepared in 0.1 M phosphate buffer (pH 7.4) for 4–5 min. The DAB reaction was stopped by adding PBS when a minimum background color appeared. The sections were then re-rinsed first in PBS and then in distilled water, ordered, mounted onto poly-lysine-coated slides, and dried overnight. Finally, they were dehydrated through ascending grades of alcohol and cleared in xylene before being covered in DPX (Sigma Life Science, USA) and a glass cover slip. The slides were observed for analysis later.

#### Cell Quantifications/Cell Counting

To quantify BrdU-positive cells, 10-μm-thick serial coronal plastic sections through the whole tectal region were prepared as described above (n = 6). BrdU-positive cells were counted on a BX50 microscope (Olympus) with an UPlanFLN 20×/10×/4× (NA0.90) objective lens. Since the size of the teleost (zebrafish) brain is slightly different among samples of the same age, we divided all sections into 10 groups along the rostro-caudal axis (six sections/groups) and calculated the mean cell number of each group in each region in the brain. This mean cell number was compared with the corresponding group of samples.

### Statistical Analysis

For statistical analyses, GraphPad Prism 5 software (GraphPad Software, Inc.) was used. Data are represented as mean and standard errors of the mean (SEM). The results acquired were analyzed by one-way ANOVA and subsequent Dunnett’s multiple comparison tests to assess the differences in seizure, latency, inflexion ratio, neurotransmitter levels, and gene expression levels between treatments and also to assess the difference in cell count at day 0 and the number of cells migrated to different regions of the brain at 15 days post-PTZ injection. For all analyses, differences between a treatment group and the equivalent negative-control group were considered statistically significant if the p-value was below 0.05 (p < 0.05).

## Results

### Seizure Analysis and Onset Latency

**Figure 2:**
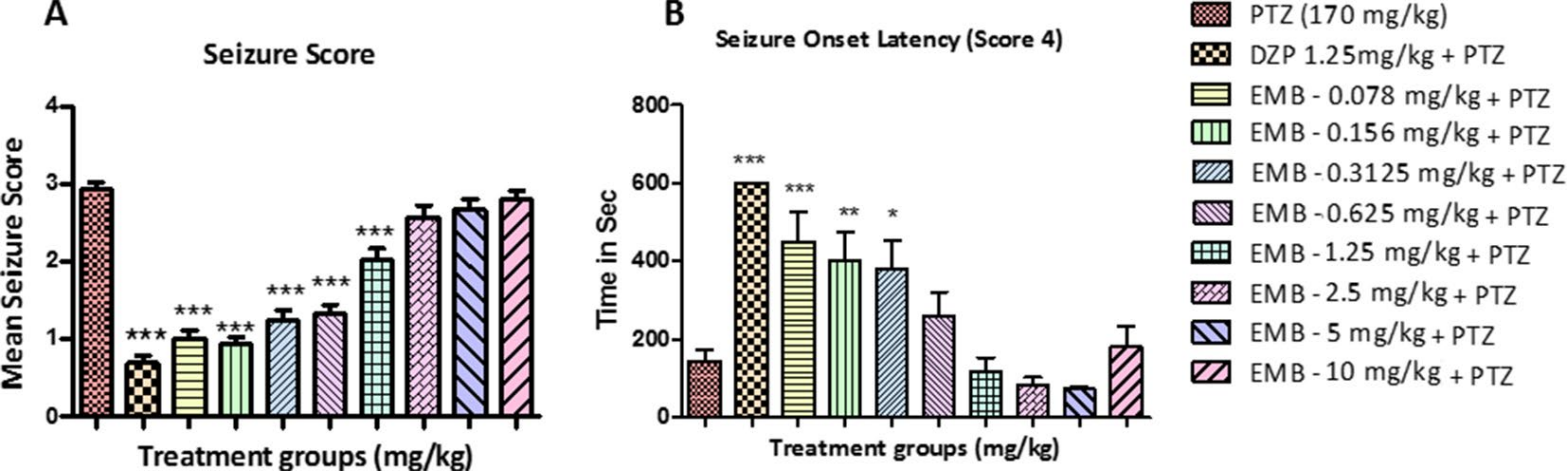
Seizure score and onset latency for epileptic seizures: **(A)** represents the effect of embelin on PTZ induced seizures in adult zebrafish. **(B)** represents onset of seizure latency score 4 for EMB and DZP treated fish when compared with PTZ treated group. Data are represented as mean ± SEM, n = 10, and statistically analyzed by one-way ANOVA followed by Dunnett’s test *P ≤0.05, **P ≤ 0.01, and ***P ≤ 0.001.

All the animals in the PTZ only treatment group showed full blown seizures up to score 4 during the 10 min recording. All the animals pretreated with EMB displayed an average seizure score of not more than 1.5, in a dose-dependent manner. It was also observed that animals treated with EMB-2.5 mg/kg to EMB-10 mg/kg (+PTZ) display seizure scores that are similar to the PTZ treated group. DZP was used as a standard drug for the epilepsy study, and it was found to suppress seizures in the DZP + PTZ treated group as shown in **Figure 2**.

Latency to seizure score 4 is the time required by the fish to reach seizure score 4 in a given time frame of 10 min. The time taken by all the animals to reach seizure score 4 from the negative control group (PTZ treated group) was 160–180 s after PTZ injection. In animals treated with DZP + PTZ, we found that the latency to reach seizure score 4 was more than 500 s. On the other hand, the onset latency was also delayed in animals treated with EMB in a dose-dependent manner. As seen in **Figure 2B**, the EMB-0.078 mg/kg to EMB-0.312 mg/kg (+PTZ) groups showed a significant increase in seizure onset latency as compared to the PTZ treated group. As the dose of the EMB increases, the activity of the drug was found to decrease and could not delay the seizure onset latency in the EMB-0.625 mg/kg to EMB-10 mg/kg (+PTZ) group.

### Locomotor Pattern

**Figure 3:**
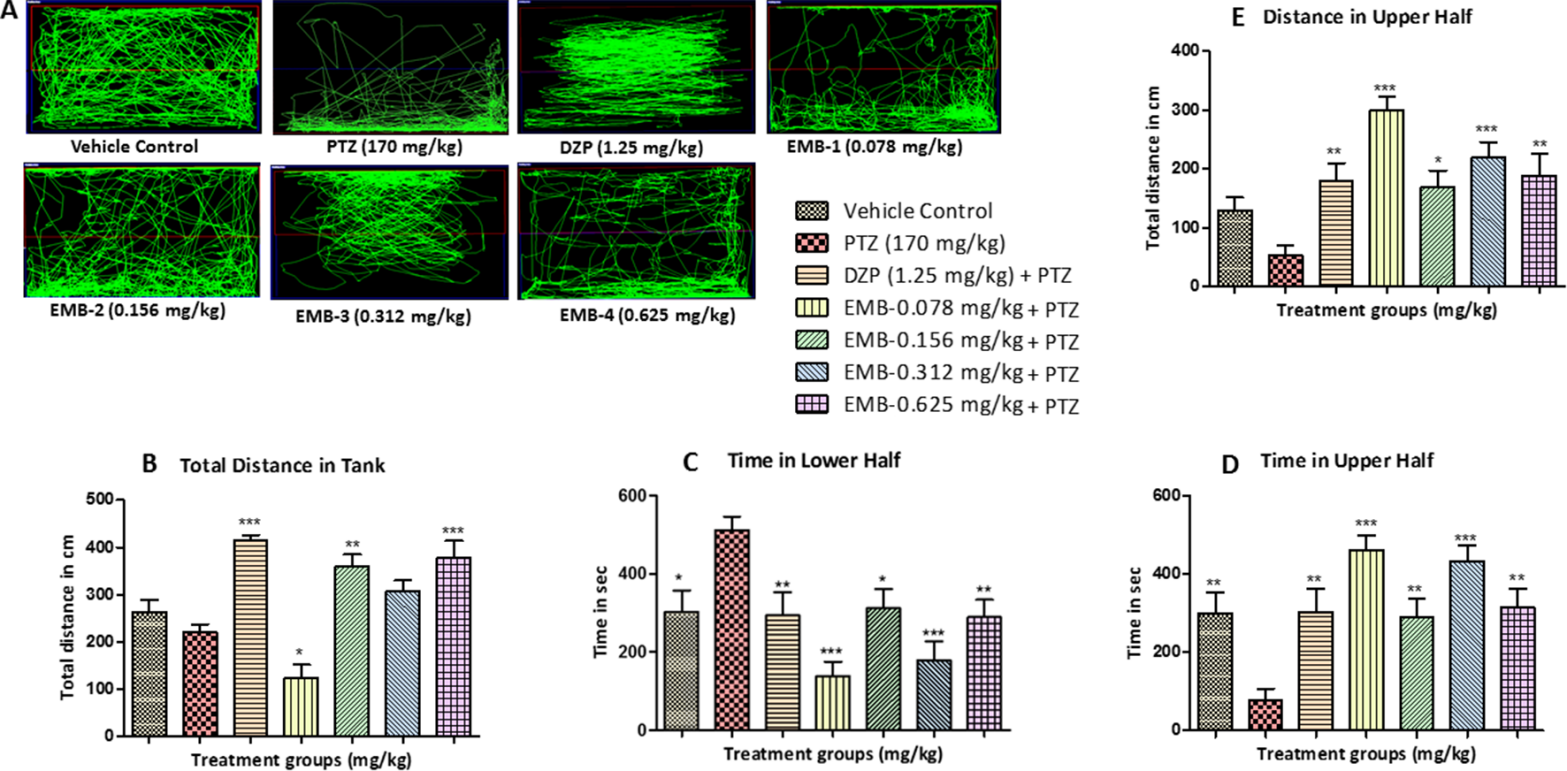
Locomotor pattern and behavior analysis of embelin treatment against pentylenetetrazole (PTZ) induced seizures: **(A)** represents the tracking pattern of locomotor behavior for the control and PTZ treated and EMB treated groups. **(B)** represents the total distance traveled by fish in each group during locomotor behavior tracking. **(C**, **D)** represent the time spent in total by each fish in the lower and upper half of the behavior tank. **(E)** represents the total distance traveled in the upper half of the tank. The data are represented as mean ± SEM, n = 10, and statistically analyzed by one-way ANOVA followed by Dunnett’s test *P ≤0.05, **P ≤ 0.01, and ***P ≤ 0.001.

A normal swimming pattern was seen in the vehicle control group, in which the tracking pattern shows swimming all over the tank. These control fish were found to spend an equal amount of time all over the tank as shown in **Figure 3A**. An involuntary, rapid movement of the body that includes a corkscrew (spiral) swimming pattern and hyperactivity was seen in the negative control group (PTZ treated) fish. PTZ provoked seizures are a spontaneous behavior which produced the tremor and jittery locomotion tracking pattern seen mainly at the bottom of the tank. The locomotor tracking pattern for most of the EMB treated groups challenged with PTZ exhibited improved tracking patterns which were almost similar to the control group. It was found from the tracking pattern of the EMB-0.156 mg/kg to EMB-0.625 groups that fish from these groups swam all over the tank, in all directions and in both the halves of the tank, without any unwanted seizure and circular movements. However, the EMB-0.078 mg/kg group tracking pattern was similar to the PTZ treated group as the fish spent more time in the lower half of the tank, with many visible circular movements. DZP was used as a standard drug and zebrafish treated with it showed a swimming pattern from the left to the right side of the tank as seen in **Figure 3A**.

The total distance traveled by the fish in the DZP, EMB-0.156 mg/kg, and EMB-0.625 mg/kg groups was significantly higher as compared to the PTZ group. The total distance traveled in the EMB-0.312 mg/kg and the control group was higher as compared to the PTZ treated group, but it was not statistically significant. But in the EMB-0.078 mg/kg treated group, the distance traveled was significantly less than the PTZ treated group, as shown in **Figure 3B**. It was found that the control fish spent nearly equal amount of time in both the halves of the tank; on the other hand, fish from the PTZ group were found to spend significantly more time in the lower half of the tank. The EMB treatment (EMB-0.312 mg/kg to EMB-0.625 mg/kg) reversed the seizure behavior, and all the fish were found to spend more time in the upper part of the tank as shown in **Figure 3**. The total distance traveled in the upper half of the tank was also found to be significantly more in all the EMB treated groups as compared with PTZ treated group, as shown in **Figure 3E**.

### Zebrafish T-Maze Test

**Figure 4:**
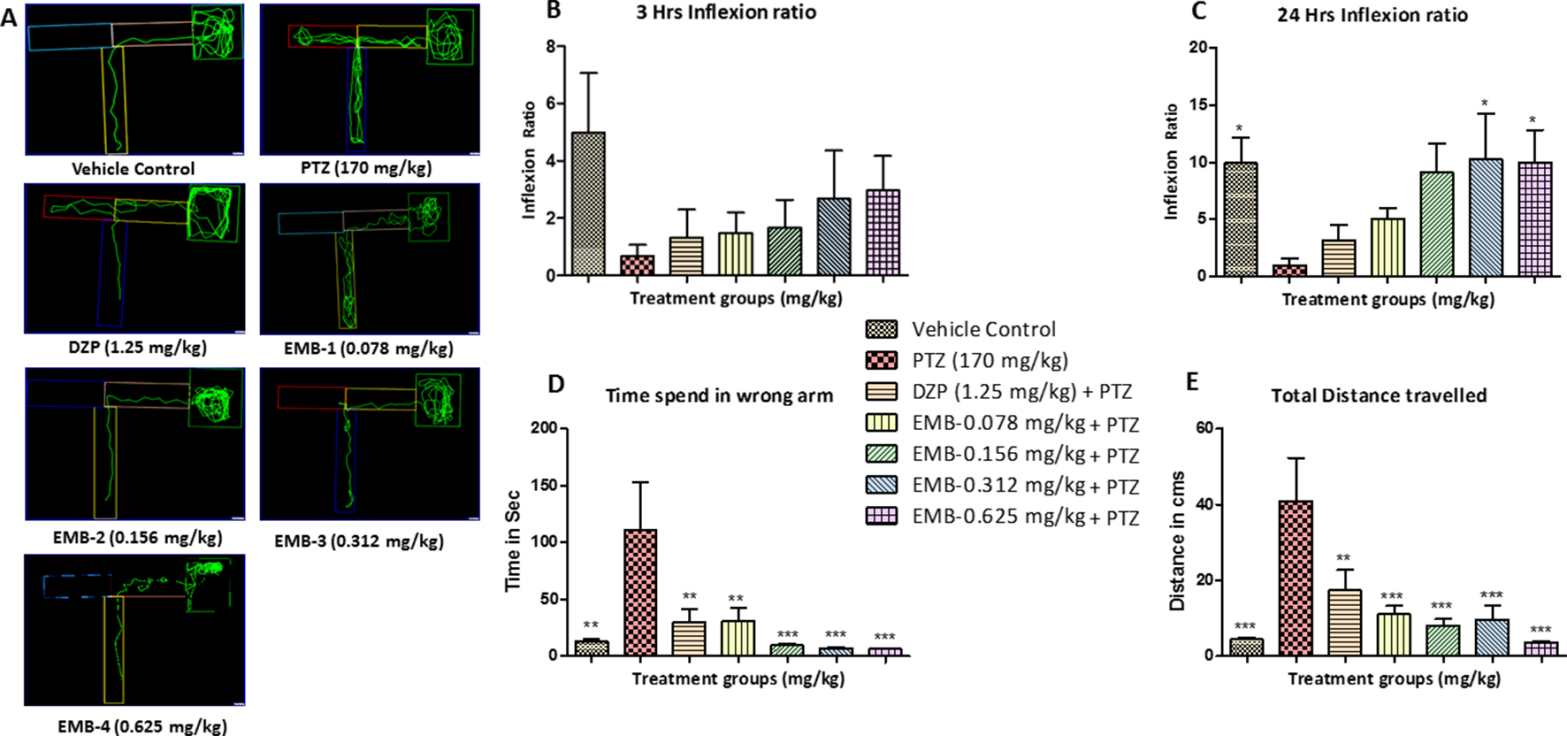
T-maze tracking pattern and behavior analysis of embelin treatment against pentylenetetrazole (PTZ) induced seizures and cognitive dysfunction. **(A)** represents the T-maze tracking pattern of locomotor behavior for the control and PTZ treated and EMB treated groups. **(B**, **C)** represent the graph plot of the inflection ratio at the 3 h and 24 h T-maze trial. **(D**, **E)** represents the time spent in the wrong arm and the total distance traveled by each fish to reach the deepest chamber of T-maze behavior tank. Data are represented as mean ± SEM, n = 8, and statistically analyzed by one-way ANOVA followed by Dunnett’s test *P ≤0.05, **P ≤ 0.01, and ***P ≤ 0.001.

In the T-maze test, fish from the vehicle control group exhibited an improved memory function in the absence of seizures. The PTZ treated group exhibited a negative effect, i.e., worsened memory function, which is depicted by a decreased inflexion ratio at both 3 and 24 h. In contrast to the PTZ treated group, EMB exhibited a better improvement of memory and showed an increased inflexion ratio in a dose-dependent manner. An increase in inflexion ratio was seen in the EMB treated group, but the results were not significant after the 3 h trial. EMB-0.312 mg/kg to EMB-0.625 mg/kg showed a significant increase in the inflexion ratio as compared to the PTZ treated group at the 24 h trial but not at 3 h. The inflexion ratio was found to be low in the DZP and EMB-0.078 mg/kg treated groups and was not found to be significant when compared to the PTZ treated-group as shown in **Figures 4B, C**.

As the inflexion ratio was low, the time required for the fish to reach the deeper chamber and the time spent in the wrong arm (left turn) was found to be higher in the PTZ group as compared to the control. The control group showed little to no repeated entry into the wrong arm and thus time spent, and distance traveled to reach the deeper chamber was found to be less. Fish from the PTZ treated group frequently failed to navigate their way to the deepest chamber and had more wrong entries into the left arm (wrong arm) before entering the deepest chamber. As the inflexion ratio of fish treated with EMB (+PTZ) was high, the time taken and distance travelled to reach the deeper chamber were less and found to be significant as compared to the PTZ treated group, which ultimately decreased the total distance traveled and time spent in the wrong arm, as shown in **Figures 4C, D**.

**Figure 5:**
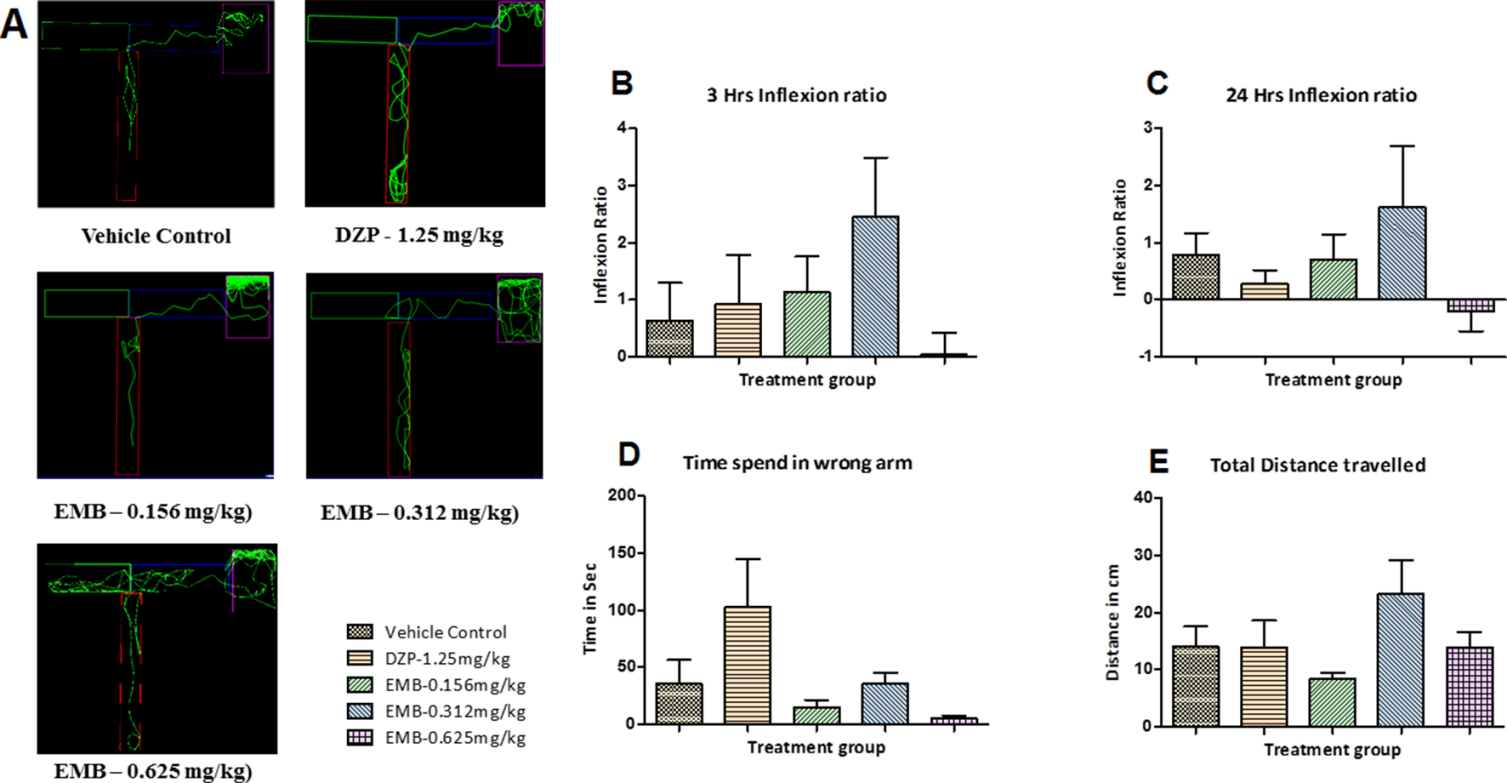
T-maze tracking pattern and behavior analysis of embelin treatment in adult healthy zebrafish: **(A)** represents the T-maze tracking pattern of locomotor behavior for the control and DZP treated and embelin (EMB) treated groups. **(B**, **C)** represent the graph plot of the inflection ratio at 3 and 24 h. T-maze trail in adult healthy zebrafish. **(D**, **E)** represent the time spent in the wrong arm and total distance traveled by each fish to reach the deeper chamber of the T-maze in adult healthy zebrafish. Data are represented as mean ± SEM, n = 8, and statistically analyzed by one-way ANOVA followed by Dunnett’s test.

The locomotor pattern of the healthy adult zebrafish in the vehicle control group was found to be normal, with a single right turn toward the deepest chamber. The locomotor pattern of adult zebrafish treated with DZP-1.25 mg/kg showed repeated back turns into the long arm and thus spent more time in the T-maze before reaching the deepest chamber. The fish treated with EMB-0.156 mg/kg and EMB-0.312 mg/kg showed similar locomotor activity as that of the control group and was found to travel toward deeper chamber, with fewer entries into the wrong arm. However, the fish treated with the EMB-0.625 mg/kg dose showed some weird behavior as they spent more time in the T-maze before entering the deepest chamber as shown in **Figures 5A–E**. On the other hand, the EMB-0.156 mg/kg and EMB-0.312 mg/kg treated groups showed a positive effect on the 3 h and 24 h inflexion ratios when compared with the control group. EMB-0.625 mg/kg failed to show increased an inflexion when compared to the control group. DZP-1.25 mg/kg showed a slight increase in the inflexion ratio at 3 h but was found to decrease the 24 h inflexion ratio when compared to the control group. The time spent in the wrong arm was found to be high in the DZP treated group. The EMB-0.156 mg/kg and EMB-0.312 mg/kg groups showed an inflexion ratio similar to that of the vehicle-treated fish as shown in **Figures 5B, C**. There was no significant difference found in total distance traveled to reach the deeper chamber in all the groups when compared with the vehicle control group, as shown in **Figure 5E**.

### Estimation of Neurotransmitters by LC/MS-MS

**Figure 6:**
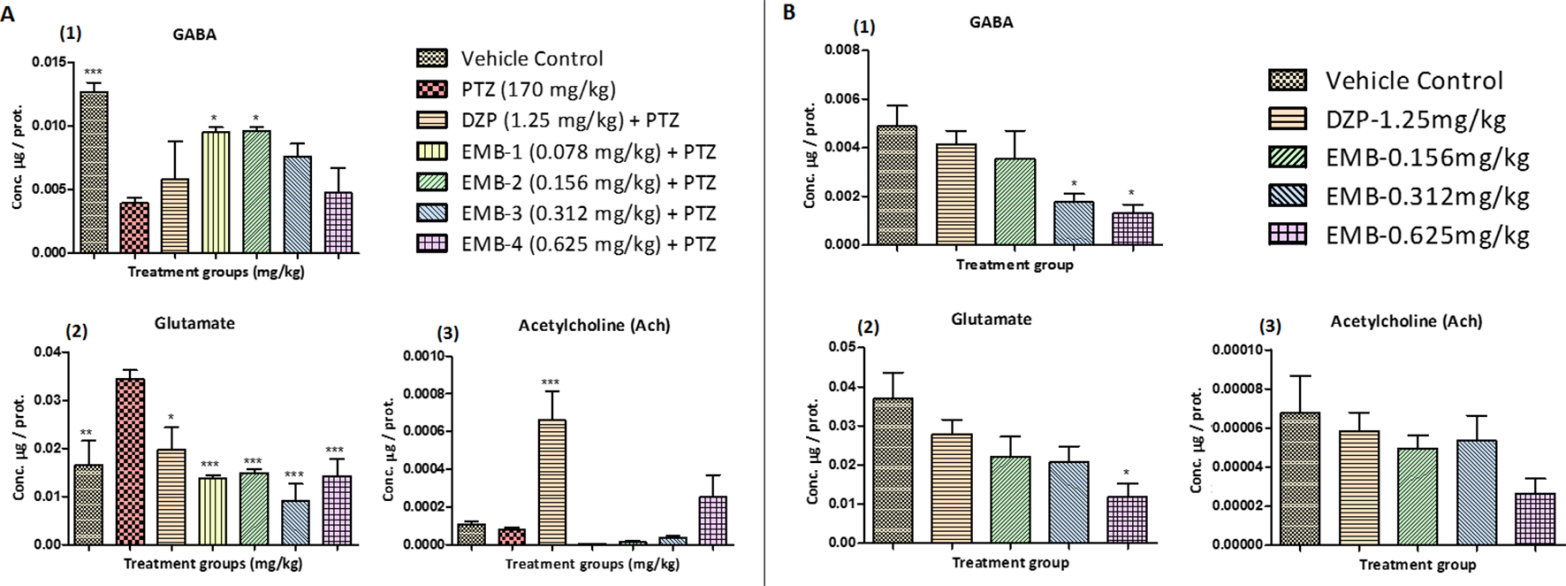
**(A)** Neurotransmitters analysis in epileptic zebrafish brains after embelin treatment and 24 h. T-maze behavior: **(A1)** represents concentration of GABA in the zebrafish brain. **(A2)** represents concentration of glutamate in the zebrafish brain. **(A3)** represents concentration of acetylcholine (Ach) in the zebrafish brain. Data are represented as mean ± SEM, n = 6, and statistically analyzed by one-way ANOVA followed by Dunnett’s test *P ≤0.05, **P ≤ 0.01, and ***P ≤ 0.001. **(B)** Neurotransmitters analysis in healthy zebrafish brains after embelin treatment and 24 h T-maze behavior: **(B1)** represents concentration of GABA in the zebrafish brain. **(B2)** represents concentration of glutamate in the zebrafish brain. **(B3)** represents concentration of acetylcholine (Ach) in the zebrafish brain. Data are represented as Mean ± SEM, n = 6, and statistically analyzed by one-way ANOVA followed by Dunnett’s test *P ≤0.05, **P ≤ 0.01, and ***P ≤ 0.001.

Neurotransmitter analysis by LC/MS-MS demonstrated elevated levels of GABA in the control group when compared to the PTZ treated groups. A significant increase in the level of GABA was found in the EMB-0.078 mg/kg—EMB-0.156 mg/kg treated groups when compared to the PTZ group. Low levels of GABA were found in the DZP and EMB-0.312 mg/kg and EMB-0.625 mg/kg treated groups as shown in **Figure 6A1**. The level of glutamate was found to be high in the PTZ treated group in comparison to the vehicle-treated group. Fish treated with EMB were protected against PTZ induced seizures due to the decreased level of glutamate. It was found that all the fish treated with EMB-0.078 mg/kg to EMB-0.625 mg/kg had a significantly lowered glutamate levels as shown in **Figure 6A2**. The level of glutamate was also found to be low in the control group and the DZP treated group. It was found that brain Ach levels were decreased in the PTZ group and were lower than the control group. DZP and the EMB-0.625 mg/kg treated groups showed an increased level of Ach as compared to the PTZ group. There was a slight increase in the level of Ach in EMB-0.312 mg/kg group, but this was not found to be significant, as shown in **Figure 6A3**.

The level of GABA in healthy zebrafish treated with EMB-0.312 mg/kg and EMB-0.625 mg/kg was found to be significantly lower when compared with the vehicle control group. However, the level of GABA was not significantly lower in EMB-0.156 mg/kg and DZP-1.25 mg/kg treated group when compared to the vehicle control group, as shown in **Figure 6B1**. The level of glutamate in the EMB-0.625 mg/kg only treated group was found to be significantly lower when compared with the vehicle control group. The level of glutamate was not significantly lower in the EMB-0.156 mg/kg, EMB-0.312 mg/kg, and DZP-1.25 mg/kg treated groups when compared to the vehicle control group, as shown in **Figure 6B2**. Similarly, there was no significant difference found in the level of Ach in all the EMB treated groups and the DZP treated group when compared with the vehicle control group, as shown in **Figure 6B3**.

### Estimation of Gene Expression by RT-PCR

**Figure 7:**
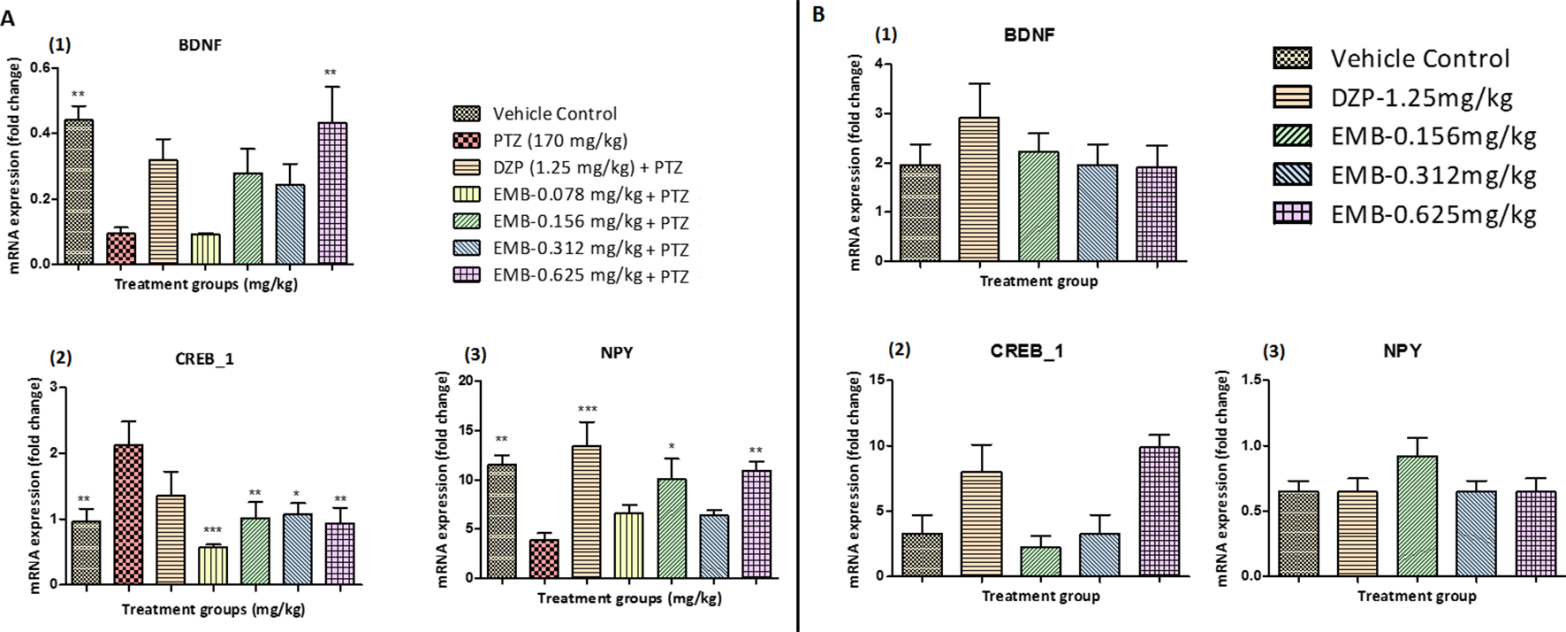
**(A)** Gene expression analysis in epileptic zebrafish brains after embelin treatment and 24 h T-maze behavior: **(A1)** represents graph plot for BDNF mRNA expression in the zebrafish brain. **(A2)** represents graph plot of CREB_1 mRNA expression level in the zebrafish brain. **(A3)** represents graph plot of NPY mRNA expression in the zebrafish brain. Data are represented as mean ± SEM, n = 6, and statistically analyzed by one-way ANOVA followed by Dunnett’s test *P ≤ 0.05, **P ≤ 0.01, and ***P ≤ 0.001. **(B)** Gene expression analysis in adult healthy zebrafish brains after embelin treatment and 24 h T-maze behavior:** (B1)** represents graph plot for BDNF mRNA expression in the zebrafish brain. **(B2)** represents graph plot of CREB_1 mRNA expression level in the zebrafish brain. **(B3)** represents graph plot of NPY mRNA expression in the zebrafish brain. Data are represented as mean ± SEM, n = 6, and statistically analyzed by one-way ANOVA followed by Dunnett’s test *P ≤ 0.05, **P ≤ 0.01, and ***P ≤ 0.001.

BDNF mRNA expression was upregulated in the control treated group as compared to the PTZ treated group. A significant elevation in the expression level of BDNF mRNA was observed in the EMB-0.625 mg/kg group. However, the DZP and EMB-0.156 mg/kg and EMB-0.312 mg/kg treated groups also demonstrated elevated levels of BDNF mRNA expression but was not significantly upregulated as compared to PTZ treated group. There was no increase in BDNF mRNA expression in the EMB-0.078 mg/kg treated group as compared to the PTZ treated group as shown in **Figure 7A1**. CREB_1 mRNA expression was down-regulated in the control group as compared to the PTZ treated group. In all the EMB treated groups, the level of CREB_1 mRNA expression was found to be significantly down-regulated as compared to the PTZ treated group, in epileptic fish. CREB_1 mRNA expression in the DZP treated group was found to be statistically insignificant when compared to the PTZ group as shown in **Figure 7A1**. NPY mRNA expression was up-regulated in the control group when compared with PTZ treated group. However, the up-regulation of NPY mRNA was improved by DZP and EMB-0.156 mg/kg and EMB-0.625 mg/kg pre-treatment as compared with the PTZ group. There were no significant differences observed with EMB-0.078 mg/kg and EMB-0.312 mg/kg pre-treatment when compared with the PTZ treated-group, as shown in **Figure 7A3**. On the other hand, there was no significant BDNF mRNA fold change observed in the EMB treated group when compared to the vehicle control group. The level of BDNF was high in the DZP treated group but was not significant when compared with the control-treated group as shown in **Figure 7B1**. The level of CREB_1 mRNA expression was found to be high in the DZP and EMB-0.625 mg/kg treated groups but was not significantly higher when compared to the control group. The level of CREB_1 mRNA expression in the EMB-0.156 mg/kg and EMB 0.625 mg/kg treated groups had no significant difference when compared with the vehicle control group as shown in **Figure 7B2**. Similarly, the EMB and DZP treated fish did not show any significant change in mRNA expression fold change of the NPY gene when compared to the vehicle control group, as shown in **Figure 7B3**.

### Molecular Docking Predictions for Embelin

**Figure 8:**
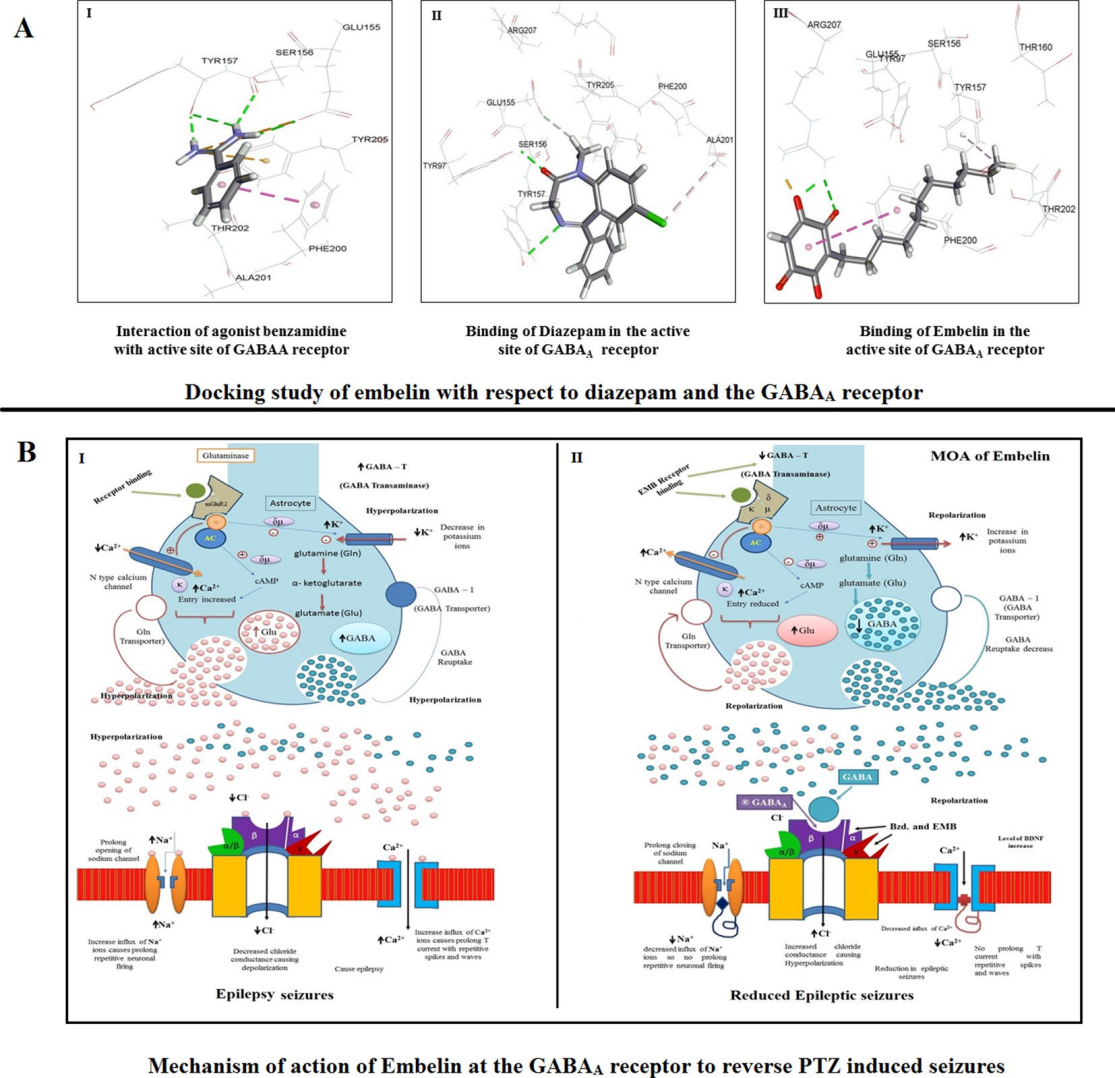
**(A)** Docking study of embelin with respect to diazepam and the GABA_A_ receptor. **(B)** Proposed mechanism of action of embelin at the GABA_A_ receptor to reverse PTZ induced seizures. 8B1—During a seizure, a prolonged opening of the voltage-gated Na^+^ channel causes an influx of sodium ions which leads to depolarization and repetitive neuronal firing. The opening of Ca2^+^ channels increases the influx of positive calcium ions causing prolonged spikes and T current waves. As there is no affinity for the GABAA receptor, the decreased Cl^−^ influx causes epileptic excitation inside the neuron cell. 8B2—EMB shows higher affinity for GABA_A_ receptor binding; it facilitates GABA mediated Cl^−^ channel opening with cell hyperpolarization and reduced seizure frequency, prolonged inactivation of the voltage-gated neuronal Na+ channel, prevented intracellular Na+ accumulation, reduced Ca2^+^ influx, and inhibited glutamate.

The docked conformation of benzamidine is shown in **Figure 8** (I). As described by Miller and Aricescu (2014), our result also showed that the phenyl ring of benzamidine forms π-stacking with Phe200 while the amidinium group interacts with Glu155, Ser 156, and Tyr 157 *via* hydrogen bonds. Furthermore, the electrostatic cation-π interaction was also observed with Tyr 205. The RMSD and CDOCKER interaction energy (CDIE) were found to be 0.85Å and −25.06 kcal/mol, respectively. Since DZP is used as a standard drug for the treatment of epilepsy, we have used it as a positive control. Therefore, we have docked DZP into the same active site of the GABA_A_ receptor. DZP also fits well in the active site, showing a CDIE of −20.28 kcal/mol. As illustrated in **Figure 8** (II), it formed hydrogen bonds with Tyr97, Tyr157, and Glu155 residues, in a similar manner to benzamidine and showed hydrophobic interaction with Ala201. When EMB was docked in the active site of the receptor, a highly favorable lower CDIE of −42.09 kcal/mol was obtained. As depicted in **Figure 8** (III), it formed a hydrogen bond and an electrostatic salt bridge with Arg207. Similarly, to the agonist benzamidine, the phenyl ring of EMB stacked with Phe200 and Tyr157. Thus, molecular docking studies indicated a good relationship between IC_50_ values and CDIE, thus supporting the biological results seen later.

### Zebrafish Brain Cell Genesis Study—BrdU Labeling

#### BrdU Labeled Cells and Proliferation Zone At Day 1

**Figure 9:**
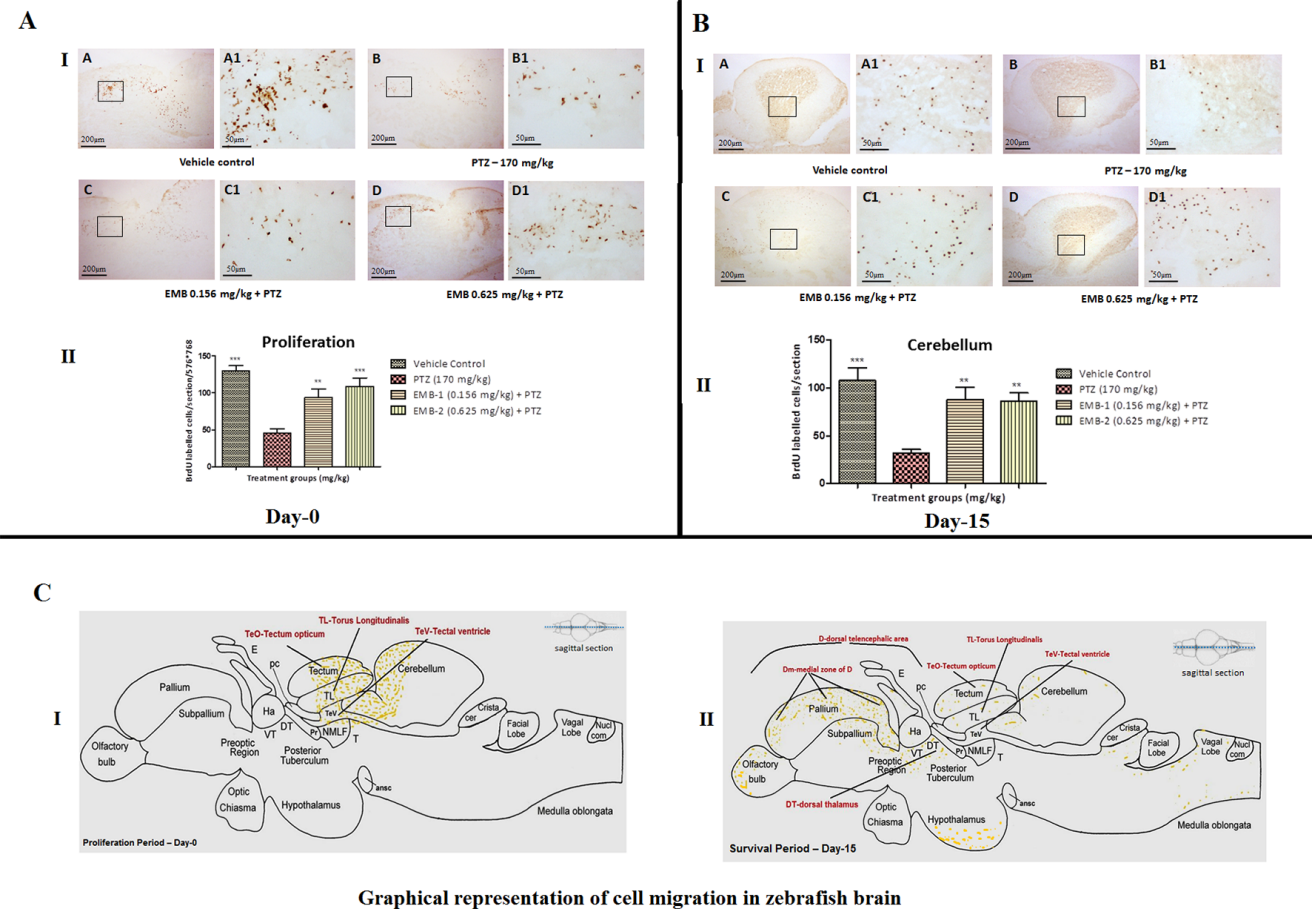
**(A)** BrdU immunohistochemistry analysis of the protective effects of embelin at the proliferation (day 0) and survival (day 15) stage against pentylenetetrazole-induced epilepsy the zebrafish brain. (A I) BrdU-positive staining revealed labeling of mitotic cells of immature neurons in the subgranular zone of the periventricular gray zone of tectum opticum with BrdU. Photomicrographs of the sagittal section of treatment groups were (A) control, (B) PTZ 170 mg/kg, (C) EMB 0.156 mg/kg + PTZ, and (D) EMB 0.625 mg/kg + PTZ. Representative photomicrographs were taken at magnifications of 40X and 200X. (II) Quantification of BrdU positive cells. Data are expressed as means mean ± SEM, n = 6, and statistical analysis by one-way ANOVA followed by Dunnett’s test ^∗∗^P < 0.01, and ***P < 0.001. (A II) BrdU immunohistochemistry analysis of the effects of embelin in improving neurogenesis and migration of cells generated during 15 days from the molecular layer to the granular layer within the dorsal zone of the periventricular hypothalamus against pentylenetetrazole-induced epilepsy the zebrafish brain. (I) BrdU-positive staining revealed labeling and migration of cells at day 15 of cell maturation and differentiation stage within the valvula cerebelli zone of cerebellum with BrdU labeling. Photomicrographs of the sagittal section of treatment groups were (A) control, (B) PTZ 170 mg/kg alone, (C) EMB 0.156 mg/kg + PTZ, and (D) EMB 0.625 mg/kg + PTZ. Representative photomicrographs were taken at magnifications of 40X and 100X. (II) Quantification of BrdU population: Data are expressed as mean ± SEM, n = 6, and statistical analysis by one-way ANOVA followed by Dunnett’s test ^∗∗^P < 0.01, and ^∗∗∗^P < 0.001. **(B)** Graphical representation of cell migration in zebrafish brain. (I) represents location of positive BrdU cells at day 0. (II) represents migration and location of positive BrdU cells at day 15.

To identify proliferation zones in the brain of adult zebrafish, the distribution of BrdU-labeled cells, as obtained after intraperitoneal injection of labeling reagent and by employing a post-administration survival time of 1 h on day 1, was analyzed. To categorize proliferation zones in the encephalon of adult zebrafish, the scattering of BrdU-labeled cells, as obtained after intraperitoneal injection of BrdU reagent and by employing a post-administration survival time of 1 h on day 1, was analyzed. At day 0, it was found that the number of BrdU positive labeled cells was significantly higher in the control group as compared to the PTZ treated group. EMB in a dose-dependent manner significantly protect neurons from PTZ seizures, such that a significant increase in BrdU positive cells was found in the EMB treated group when compared to the PTZ treated group. It was also found that, at day 0, newborn cells tagged with the BrdU label were found to be originating from the tectum opticum (TeO), torus longitudinal (TL), tectal ventricle (TeV), and cerebellum region of the zebrafish brain as shown in **Figure 9A**.

#### Distribution of Positive BrdU-Labeled Cells After 15 Days of Survival

**Figure 10:**
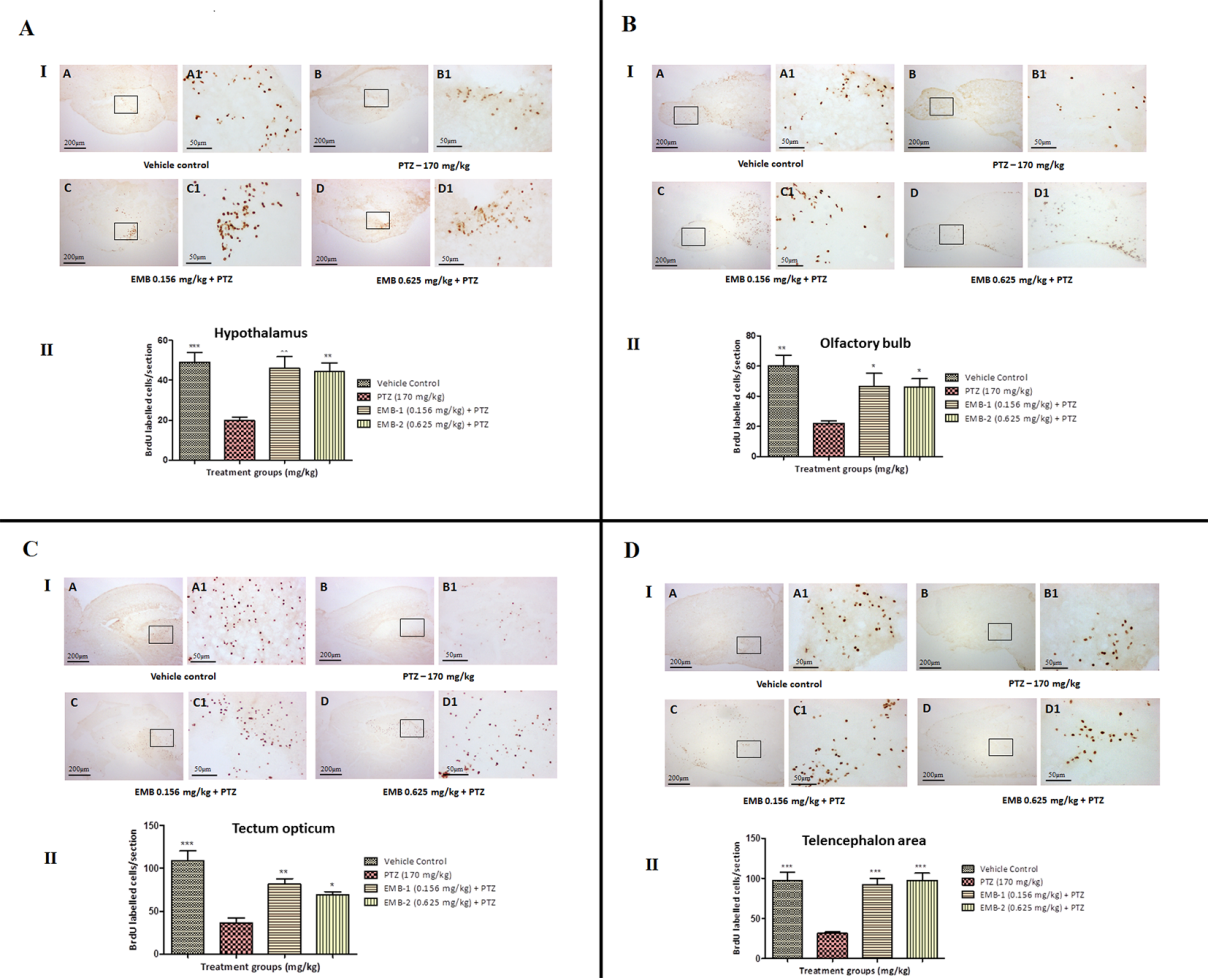
BrdU immunohistochemistry analysis of the effects of embelin in improving neurogenesis and migration of cells generated during adulthood from the molecular layer to the granular layer within the molecular zone of the hypothalamus, the internal cellular layer of the olfactory bulb of the periventricular gray zone of TeO, and the medial zone of the dorsal telencephalic area against pentylenetetrazole-induced epilepsy the zebrafish brain. (I) BrdU-positive staining revealed labeling and number of migration of cells at day 15 of maturation and differentiation stage at the molecular zone of each section of zebrafish brain with BrdU labeling. Photomicrographs of the sagittal section of treatment groups were **(A)** control, **(B)** PTZ 170 mg/kg alone, **(C)** EMB 0.156 mg/kg + PTZ, and **(D)** EMB 0.625 mg/kg + PTZ. Representative photomicrographs were taken at magnifications of 40X and 100X. (II) Quantification of BrdU population: Data are expressed as mean ± SEM, n = 6, and statistical analysis by one-way ANOVA followed by Dunnett’s test *P < 0.05, **P < 0.01, and ***P < 0.001.

To find out a possible survival and migration pattern of the new cells after EMB and PTZ administration, the distribution of BrdU-positive cells was analyzed in five brain regions olfactory bulb, cerebellum (rhombencephalon), TeO (mesencephalon), telencephalic area (telencephalon), and hypothalamus (diencephalon). As seen, most of the cells from molecular layers migrated toward the granular layer and were found to be high in control and EMB treated brains when compared to PTZ treated fish brains. The number of positive BrdU cells found in the olfactory bulb, cerebellum, TeO, telencephalic area, and hypothalamus was significantly higher in the control and EMB treated group as compared to PTZ treated the group as shown in **Figure 10**.

## Discussion

A common method for inducing animal epilepsy are chemoconvulsants, which are chemical agents that can produce seizures (Choo et al., 2018). In a dose deciding study, we found that three doses of EMB (0.625 mg/kg, 0.312 mg/kg, and 0.156 mg/kg) had significant seizure protection activity. Further increasing the EMB dose reduced its seizure protection activity. This suggests that EMB has a small therapeutic window, which corroborates findings in a rodent study (Bhuvanendran et al., 2018). PTZ treated fish showed anxiety behavior as they mainly resided at the bottom half of their tank, in contrast to control fish which spent equal amounts of time in both halves. EMB treatment of up to 0.625 mg/kg reduced PTZ-induced seizures and treated fish exhibited normal swimming movements consisting of repeated short swims.

The T-maze is a standardized learning and memory paradigm (Hamilton et al., 2016) whereby zebrafish attempt to reach a deeper chamber using their spatial memory for a spacious and favorable environment (Lamb et al., 2012). The current study suggests that epileptic zebrafish treated with EMB had the ability to successfully learn, navigate, and discriminate between the wrong and right arms to reach the deeper chamber. A similar effect of EMB was also observed in healthy adult zebrafish. The 3 and 24 h inflexion ratios showed a significant increase in memory function in the EMB-0.312 mg/kg and EMB-0.625 mg/kg groups as compared to the PTZ treated group. In the zebrafish brain, the olfactory bulb in the telencephalon is responsible for governing egocentric navigating myopia that helps the fish reach the deeper chamber (Lindsey et al., 2014). During an epileptic episode, the connection between the olfactory bulb and the telencephalon is likely impaired, and this may affect the learning and memory ability of the fish. But when the fish is pre-treated with EMB, it prevents the fish from becoming epileptic and also increases its learning ability, which leads to the increased 24 h inflexion ratio.

Abnormal neurotransmitter levels are related to many neural diseases including epilepsy (Levin and Cerutti, 2009). GABA is the primary neuroinhibitor in the CNS (Kaila et al., 2014) and plays a well-studied and important role in epilepsy as well as learning and memory (Luo et al., 2011). In the current study, we reported increased GABA levels in all EMB groups, proving the role of GABA in learning and memory. EMB may have some ability to suppress Ca^2+^ influx and increase chloride conductance as epileptogenesis is aided by increased Ca^2+^ influx (Zamponi et al., 2010), reduced chloride conductance, and decreased GABAergic presynaptic inhibition (Werner and Covenas, 2011). Extremely augmented excitatory glutamate release is one of the leading causes of epileptic seizures (Rowley et al., 2012). Glutamate toxicity is associated with neuronal death in neurodegenerative disorder cases (Guerriero et al., 2015), and glutamate also affects memory and learning (Izquierdo and Medina, 1997). In the present study, a lower level of glutamate was found in all groups as compared to the PTZ treated group. PTZ acts at the GABA_A_ receptor and reduces chloride conductance (Zhang et al., 2017), leading to glutamate excitation (Banote et al., 2013). Fish getting repeatedly lost in the wrong arm of the T-maze in the PTZ treated group shows a relation between memory loss and glutamate toxicity, which was reversed by EMB treatment. Modulating memory formation is a key role of Ach, which also alters neuron excitability, prompts synaptic plasticity, and controls the firing of groups of neurons (Picciotto et al., 2012). An increase in Ach levels was also observed in the EMB treated groups but was not significant when compared to the PTZ treated group. DZP is reported to be an acetylcholinesterase (AChE) inhibitor and thus the level of Ach was high in the DZP treated group as the level and duration of Ach in the brain is increased (Lundgren et al., 1987).

BDNF has a remarkable role in synaptic plasticity, survival, and differentiation of neurons in the brain (Roopra et al., 2012) as well as increasing the persistence of short- and long-term memory storage (Bekinschtein et al., 2008). It has been discovered that BDNF is linked to the inception of epileptogenesis, and the epileptic condition can be avoided by BDNF upregulation (Binder, 2004). In the present study, we found that BDNF mRNA expression was down-regulated in the PTZ treated group when compared with the control but increased in the EMB treated groups, indicating memory improvement, neuronal survival, and differentiation of new growing neurons (Choi et al., 2018). CREs are promoters that are phosphorylated to generate epilepsy (Zhu et al., 2012). Studies in rodents show that a decrease in CREB levels has a 50% chance to decrease seizure episodes (Zhu et al., 2012) and that CREB_1 modulates synaptic plasticity for the intrinsic excitability of the neurons (Benito and Barco, 2010). CREB_1 mRNA expression was high in PTZ treated fish, confirming its role in epileptogenesis. In all the EMB treated groups, CREB_1 mRNA expression was found to be down-regulated, which indicates that EMB helps in reducing epilepsy and enhances memory. NPY has an important role in regulating physiological processes such as various brain events, memory, and learning (Kas et al., 2005). Earlier rodent studies reported that chronic spontaneous seizures are reduced by NPY in a temporal lobe epilepsy model of rats (Noe et al., 2010) and that high levels of brain NPY are crucial for memory and learning (Gøtzsche and Woldbye, 2016). The level of NPY mRNA expression in the PTZ treated group was significantly downregulated as compared to the control group. However, EMB treated fish showed increases in NPY expression, indicating that it plays a crucial role in modulating neurotransmitters, increasing neuronal growth, and preventing epilepsy in zebrafish brains (Vezzani and Sperk, 2004).

Docking studies are mainly performed to determine the affinity of a particular compound for a receptor or a similar molecule (Meng et al., 2011). We found that EMB has a high affinity for the benzodiazepine active binding site of the GABA_A_ receptor at the β3 subunit. During epileptic seizures, the extra release of glutamate and increased breakdown of GABA causes neuronal firing and seizure episodes (Engel, 2013). By decreasing GABA release, there is much less binding of GABA to the GABA_A_ receptor, decreasing the influx of Cl^−^ ions and hence causing excitability of the neuron cell. EMB’s high affinity toward the GABA receptor may help in GABA release and in reducing cellular excitability by prolonged inactivation of voltage-gated neuronal Na^+^ channels, preventing intracellular Na^+^ accumulation and inhibiting high-frequency discharge. A reduction in Ca^2+^ influx and inhibition of glutamate may also take place after EMB treatment (Eraković et al., 2000). As EMB binds to the benzodiazepine G-protein coupled receptor at the α or γ subunit, it facilitates GABA mediated Cl^−^ channel opening with cellular hyperpolarization and a reduction in seizure frequency (Benarroch, 2007). This could serve as a clue toward unraveling the precise mechanism of EMB against epilepsy and related cognitive dysfunction.

Generation of neurons from neuronal stem cells is known as the process of neurogenesis (Fuchs and Gould, 2000). During neurodegeneration, the subgranular zone (SGZ), which is the part of the hippocampus, the dentate gyrus, and the subventricular zone (SVZ) are active continuously (Ming and Song, 2011). Proliferation and neurogenesis are not clearly understood in adult zebrafish. There is only one study that describes the proliferative zones and the migration of cells in the telencephalon, preoptic region, thalamus, hypothalamus, midbrain, and cerebellum (Kaslin et al., 2008). A similar result was found in the present study, where newborn cells at day 0 were found to be tagged in abundance with BrdU at TeO, TL, TeV, and the cerebellum region of the brain. The number of BrdU tagged cells was found to be high in control and EMB treated groups as compared to the PTZ treated group. Epileptic seizures induced by PTZ causes increased oxidative stress and inflammation, which leads to neuronal death in the brain (Naseer et al., 2014).

Labeling of dividing cells with the BrdU thymidine analog provides insight into EMB’s effect on cell proliferation and migration after a 15 days survival period (Grandel et al., 2006). Mitotic activity was specifically pronounced in the olfactory bulb, hypothalamus, telencephalic area (dorsal telencephalon), preoptic area of the diencephalon, optic tectum of the mesencephalon, TL, and in all three cerebellum subdivisions (Zupanc et al., 2005). In the zebrafish optic tectum, there was a lack of evidence for a long-distance migration of the new cells from their proliferation zones. Among all brain regions, the different subdivisions of the cerebellum demonstrated a maximal number of mitotic cells in control and EMB treated zebrafish. A similar result has been reported in other teleosts including guppies and the brown ghost (Rubenstein and Rakic, 2013). As the administration of PTZ damages newly born cells at the s4 phase of mitosis, the number of cells which migrated to different parts of the brain was significantly lower. Evidence of PTZ causing neurodegeneration was also previously observed (Park et al., 2006). In spite of extended neuronal growth after the epileptic episode, studies have shown that an aberrant neuronal circuit causes more severe damage to the brain structure. As the fish were pre-treated with EMB in this study, they protected the brain cell during the PTZ insult and caused less epileptic episodes and more neuronal survival. On the other hand, as the newly tagged BrdU cells in PTZ treated group were damaged after the PTZ insult, the numbers of cells at day 0 and day 15 were found to be less as compared to the control and EMB treated groups.

## Conclusion

EMB is reported to have various activities such as being an anti-oxidant and anti-inflammatory and can cross the BBB. Findings from the current study demonstrate that EMB can also suppress seizure-like behavior and improve cognitive function in zebrafish. The docking study showed that EMB has a higher affinity toward the GABA_A_ receptor. In addition, the current study also explored the basic mechanism of EMB, its possible site of receptor binding and its ability to promote cell migration and differentiation. The study conducted on healthy zebrafish with only EMB treatment showed similar memory behavior and no alteration in gene expression level affecting the memory status of the fish. This implicates that EMB does not interfere with the normal functioning of body processes and has no adverse effect. T-maze data, behavioral study, immunohistochemistry staining, and biochemical analysis supported the observed effect of EMB. Herein, we suggest that EMB could be a promising candidate against epilepsy induced learning and memory dysfunctions. Further investigations utilizing different seizure models are warranted and will strengthen the potential of EMB toward clinical translation. Current findings shed light on the utilization of plant-based natural compounds against epilepsy and related cognitive impairment. These will overcome the limitations of mainstream AEDs and will be an economical option as well.

## Data Availability Statement

The datasets generated for this study are available on request to the corresponding author.

## Ethics Statement

The experimental protocol was approved by the Monash Animal Research Platform (MARP) Animal Ethics Committee, Monash University, Australia (MARP-2015-084).

## Author Contributions

UK performed most of the experimental procedure along with analysis of the results and writing of the manuscript. BC helped in performing behavior recording, data analysis, writing of the manuscript, and proofreading. NA contributed to the molecular docking study. YK contributed to designing the gene expression study and result analysis. IO contributed to LC-MS/MS study. MS conceptualised the idea, contributed to study design, result interpretation, analysis, manuscript writing, and proofreading.

## Funding

This research work was supported by the eScience Fund of Ministry of Science, Technology, and Innovation (MOSTI), Malaysia (Grant No. 06-02-10-SF0250).

## Conflict of Interest

The authors declare that the research was conducted in the absence of any commercial or financial relationships that could be construed as a potential conflict of interest.
